# Nitrogen-Dopped Ordered Mesoporous Carbon Anchored Pd Nanoparticles for Solvent Free Selective Oxidation of Benzyl Alcohol to Benzaldehyde by Using O_2_

**DOI:** 10.3389/fchem.2019.00458

**Published:** 2019-06-27

**Authors:** Hongbing Song, Zong Liu, Hengjun Gai, Yongjie Wang, Lin Qiao, Caiyun Zhong, Xiangyang Yin, Meng Xiao

**Affiliations:** State Key Laboratory Base for Eco-Chemical Engineering in College of Chemical Engineering, Qingdao University of Science and Technology, Qingdao, China

**Keywords:** nitrogen-doped ordered mesoporous carbon, hydrothermal self-assembly, benzyl alcohol, selective oxidation, benzaldehyde

## Abstract

Introducing electron-rich nitrogen atoms to ordered mesoporous carbons (OMC) as supports for noble metal catalysts, not only improves the hydrophilic properties of a mesoporous carbon surface, but also enhances the coordination and binding abilities of metal ion. In the present work, nitrogen-doped ordered mesoporous carbons (NOMCs) were successfully fabricated via a facile hydrothermal self-assembly. The prepared NOMCs were characterized through powder X-ray diffraction (XRD), Raman spectroscopy, transmission electron microscopy (TEM), X-ray photoelectron spectroscopy (XPS), and nitrogen adsorption-desorption isotherm. The analyses demonstrated that the NOMCs prepared at a pyrolysis temperature of 750°C possessed an ordered 2D hexagonal mesoporous structure, a high graphitization degree, large surface area, and a well-distributed pore size. In particular, NOMCs could anchor Pd nanoparticles uniformly because of the introducing N atoms with strong electronegativity, which were selected as efficient catalysts for the partial oxidation of benzyl alcohol to benzaldehyde. Approximately 24.63% conversion with 85.71% selectivity to benzaldehyde was obtained without using any solvent by molecular O_2_ oxidation. Most importantly, the TOF value of the catalyst in the reaction system was up to 8698 h^−1^. After five runs reaction, TOF and selectivity of the catalyst remained essentially same. Hence, the proposed catalyst has a potential engineering application value.

## Introduction

The partial oxidation of aromatic alcohols plays a key role in organic chemistry, and their corresponding aldehydes are essential building blocks for the industrial synthesis of other chemicals (Homma and Kitaoka, 2016; Zhu et al., 2017). However, traditional oxidation processes were performed in solvents by using toxic or corrosive stoichiometric oxidants and produced large amounts of wastes (Watanabe et al., 2015). Thus, some highly effective and environmentally friendly methods have been developed to carry out the oxidation reaction (Dai et al., 2016). For instance, the use of oxygen (O_2_) is preferred at atmospheric pressure from economic and green chemistry viewpoints because O_2_ is relatively cheap and produces only water as a byproduct in the reaction (Shanahan et al., 2017). Moreover, traditional catalyst systems require the use of solvents. Hence, the catalysts operated in a solvent–free should be explored to improve environmental friendliness. As a result, a nanocarbon–based catalyst has shown potential as an alternative to meet the requirements of sustainable chemistry (Kuang et al., 2010; Luo et al., 2012, 2014).

Carbon materials are widely used as support because of their advantageous features, including large specific surface area, porous structure, low cost, environmental friendliness, and stability (Schrettl et al., 2016; Eftekhari and Fan, 2017; Hu et al., 2017; Xiao et al., 2017a; Zhang et al., 2017). Among them, mesoporous carbon as a catalyst support has been widely explored because its narrow pore size distribution provides a larger reaction space and shortens mass and heat transfer path for substrates and products, thereby improving their catalytic performance. In comparison with ordinary mesoporous carbon materials, the pore size distribution of ordered mesoporous carbons (OMCs) is uniform, and the channel arrangement is regular (Luo et al., 2016; Wang and Zhao, 2019). In this case, the interaction with analytes can be maximized, and fast mass transport can be ensured (Walcarius, 2012; Maluta et al., 2018). These unique properties lead to numerous possible applications in the field of separation, adsorption, electrochemistry and catalysis (Wang et al., 2016, 2018; Libbrecht et al., 2017; Liu et al., 2017; Xiao et al., 2017b; Chen et al., 2018; Gao et al., 2018; Xu et al., 2019; Zhu et al., 2019).

Moreover, the adsorption properties of OMCs can be significantly enhanced by introducing nitrogen atoms into its structure (Wu et al., 2012; Shen and Fan, 2013; Wang R. et al., 2014a; Bayatsarmadi et al., 2015; Cui et al., 2017). That is because the lone–pair electron from N can activate *p*–electrons in carbon materials (Zhao et al., 2013), thereby improving the hydrophilic properties of the mesoporous carbon surface and the electron–donor properties of the carbon matrix (Wu and Zhao, 2011; Liu Z. et al., 2012; Wang J. et al., 2014). In particular, the metal particles attached to the N-doped carbon are not easily detached, and as a catalyst, it has a stable nanostructure to effectively catalyze oxidation reaction (Zhang et al., 2013; Song et al., 2017).

In terms of preparation, the typical method to prepare NOMCs is the template method. Moreover, the template can be removed by chemical etching or pyrolysis. Chemical etching is often used to remove inorganic templates (such as SiO_2_, Al_2_O_3_, or zeolite), which have high temperature resistance and water insolubility, but can react with acids and bases (such as HCl, NaOH, or HF) to form soluble substances or gases. Chemical etching is usually carried out after carbonization. Soft-templates such as F127 can be pyrolyzed at high temperatures. Due to the conditions of pyrolysis and carbonization are consistent, the preparation process can be simplified, and no additional acid/alkali solution is consumed, resulting in less pollution. N-doping materials can be obtained via two common routes: retreatment of the pre–synthesized OMC with N–containing materials, such as NH_3_, and one–step direct pyrolysis N–contained carbon materials. The latter has a higher doping efficiency and is environmentally friendly and convenient in aqueous solution (Liu Z. et al., 2012; Deng et al., 2016).

In our study, NOMCs were successfully fabricated by introducing melamine resin to modify phenolic resin in which the –NH– group was connected to carbon atoms to form an orderly mesoporous structure and function as an effective support to immobilize Pd nanoparticles. The synthesized NOMCs were characterized through powder X–ray diffraction (XRD), X–ray photoelectron spectroscopy (XPS), transmission electron microscopy (TEM), and Brunauer–Emmet–Teller (BET) method. The results illustrated that the samples exhibited an ordered mesoporous structure. The BET surface area could exceed 600 m^2^/g, and the pore volume could be up to 0.3 cm^3^/g. The degree of graphitization in NOMCs was implied by Raman spectroscopy and wide–angle XRD. NOMCs supported by Pd nanoparticles were employed as catalysts for the aerobic oxidation of benzyl alcohol with molecular O_2_ as an oxidant. The catalytic activity and stability of NOMC catalyst were further studied.

## Experimental Section

### Materials

Triblock poly(ethylene oxide)–b–poly(propylene oxide)–b–poly(ethylene oxide) copolymer Pluronic F127 (EO_106_ PO_70_ EO_106_, M_av_ = 12600) was purchased from Sigma–Aldrich Corporation. Other chemicals resorcinol, hexamethylenetetramine (HMT), melamine and ammonia were purchased from Shanghai Aladdin Bio–Chem Technology Co., LTD. All chemicals were used as received without further purification.

### Preparation of NOMCs

NOMCs were synthesized by modified aqueous self–assembly route with ampliphilic triblock copolymer Pluronic F127 as a template (Ma et al., 2018). Typically, 1.1 g of resorcinol, 2.2 g of Pluronic F127, 0.7 g of HMT, and 2.0 mL of aqueous ammonia (28%) were mixed with 54 mL of deionized water. After 1 h stirring at room temperature, 0.375 g of melamine and another 0.4 g of HMT were added into the mixture. The final reactant mass ratio of resorcinol/F127/HMT/melamine was 3/6/3/1. The obtained dark solution was further stirred for 24 h at 80°C. The reddish black solid products were cooled to room temperature and collected by sedimentation and filtration. Afterwards, the solid products were washed by water and air–dried at 80°C overnight. Finally, the as–synthesized samples were calcined in a tubular furnace under nitrogen atmosphere at 2°C·min^−1^ up to a certain temperature and further treated for another 2 h. Then the samples of NOMC–x–y were obtained under different conditions, where x denoted the molar ratio of melamine to resorcinol, y denoted the calcined temperature.

### Preparation of Pd/NOMC Catalysts

Pd nanoparticles supported on an NOMC (zPd/NOMC−0.3–750, z denoted the mass percentage of Pd, 0.3 denoted the molar ratio of melamine to resorcinol, 750 denoted the calcined temperature°C) were prepared through adsorption–reduction method. Typically, 144 μL of 0.1 M H_2_PdCl_4_ and 300 mg of NOMC−0.3–750 were dissolved in 20 mL of deionized water. This mixture was stirred for 10 h at 60°C and cooled to room temperature. Then, the mixture was ultrasonicated for 10 min and NaBH_4_ solution (5 equivalent to Pd used, 0.2 M) was added to the solution by drop. The precipitate was centrifuged and washed with deionized water. The resulting Pd catalyst was dried in an oven at 40°C overnight, and the catalyst 0.5Pd/NOMC−0.3–750 containing 0.5 wt.% Pd was obtained. To obtain 1Pd/NOMC−0.3–750 (1 wt.% Pd) and 2Pd/NOMC−0.3–750 (2 wt.% Pd), 288 μL and 576 μL of 0.1 M H_2_PdCl_4_ were added during the preparation, respectively.

### Characterization

The structures of as–synthesized carbons were determined by small and wide–angle X–ray diffraction (SXRD and WXRD) measurements on D–MAX Advance using Cu Kα radiation (1.5406 Å) at an operating voltage of 45 kV. Transmission electron microscopy (TEM) images and energy dispersive X-ray analysis (EDX) images were carried out using the JEM−1200EX (JEOL) operated at an accelerating voltage of 200 kV. The samples were dispersed into ethanol under sonication for 3 min and deposition on a carbon–coated copper grid before the measurement. Raman spectroscopic investigations were conducted on a HORIBA Scientific Raman system, with a LabRAM HR Evolution spectrometer. Nitrogen adsorption–desorption isotherms were measured using an ASAP 2020 volumetric adsorption analyzer with the 99.998% purity of N_2_. Before adsorption measurement, the samples were degassed for 10 h at 120°C. The specific surface areas were calculated using the Brunauer–Emmett–Teller (BET) equation in the relative pressure (P/P_0_) range of 0.05–0.2. The pore–size distribution curves were obtained by analysis of the adsorption branches of the isotherms. The total pore volumes were estimated from the amount adsorbed at a relative pressure of P/P_0_ > 0.995. The X–ray photoelectron spectroscopy (XPS) was collected on an ESCALAB 250 XI electron spectrometer from Thermo Scientific with an Al K_α_ X–ray source (hν = 1486.71 eV, 5 mA, 15 kV) incident beam in a N_2_ atmosphere, and the calibration was performed by setting the C 1s peak at 284.5 eV.

### Catalytic Oxidation of Benzyl Alcohol

In a typical oxidation, 5 mL of benzyl alcohol and 20 mg of NOMC catalyst were added into a 25 mL round bottom flask reactor equipped with a magnetic stirrer and an air inlet tube. The reaction was performed at 160°C in an oil bath at a stirring rate of 1000 rpm. Molecular oxygen was conducted into the reaction mixture and controlled by a flowmeter at a constant flow rate (20 mL·min^−1^). After the reaction, the liquid phase was collected by filtration. Then, the mixture was analyzed by gas chromatography (GC, SP2100) using a flame ionization detector equipped with a DB−5 capillary column (30 m × 0.25 mm × 0.25 μm). On the other side, gas chromatography mass spectrometer (GC–MS, Agilent 7000C) was applied to analyze the component of the product. The turnover frequency (TOF) was based on the surface Pd molar number obtained by H_2_ titration and calculated according to the following formula.

$$\begin{array}{l} \text{TOF}=\frac{reacted\;benzyl\;alcohol\;\left(mol\right)}{amount\;of\;the\;active\;sites\;\left(mol\right)\;\times \;reaction\;time\;\left(h\right)\;} \end{array}$$

## Results and Discussion

### Characterization of NOMCs

Unlike the conventional soft-template and hard-template methods, precursor polymerization and package template were performed simultaneously (Schrettl et al., 2016; Gai et al., 2018). NOMCs were synthesized via simple hydrothermal self–assembly by using resorcinol/F127/HMT/melamine. HMT was used as the slow release source of formaldehyde to control the kinetic of polymerization. Polymerization and self–assembly processes were carried out in parallel without pre–polymerization. Ammonia acted as a catalyst for resorcinol and formaldehyde polymerization, which was similar to the evaporation–induced self–assembly (EISA) pathway. Considering the high reactivity of amino groups and the high nitrogen content, melamine was chosen as a nitrogen source. Doping of nitrogen atoms could improve the surface polarity of the carbon matrix and introduce the anchored sites into the carbon skeleton (Li and Xue, 2014).

In determining the influence of melamine and temperature, the mass ratio of resorcinol/F127/HMT was held constant at 1:2:1, and a series of samples with different amount of melamine and different carbonization temperature were prepared. The molar ratio of melamine to resorcinol was changed from 0 to 0.9, and the carbonization temperature was held from 550 to 850°C.

**Figure 1:**
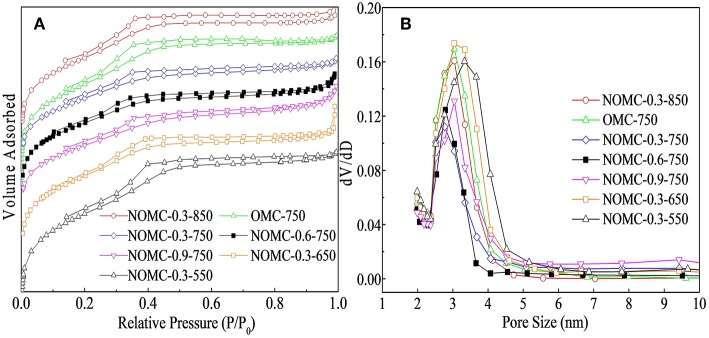
N_2_ sorption isotherms **(A)** and pore–size distribution plots **(B)** of OMC and NOMCs.

Nitrogen adsorption/desorption measurements were carried out at 77 K to analyze the textural properties of NOMCs and OMC. The N_2_ sorption isotherms and the pore size distribution curves of NOMCs and OMC are shown in Figure 1. The adsorption and desorption branches of all of the samples formed a Type IV isotherm with a small H2 hysteresis loop, corresponding to the 2D hexagonal p6m mesoporous structure of carbon materials. The two branches were not tight at a low pressure, indicating the presence of a certain proportion of micropores, which could be confirmed from the textural parameters of the samples in Table 1. The samples obtained at high pyrolysis temperatures possessed a large BET surface area because the removal of the PEO segments produced additional micropores, resulting in a more developed pore structure (Liu et al., 2010). High nitrogen doping and high order degree could not usually coexist. As the molar ratio of melamine increased, the structural parameters of the samples were obviously affected, and this observation was related to the reduction of the order degree due to the collapsed pores. The diameter of the primary mesoporous pore was calculated by the cylindrical program with ~3.1 nm, but this method slightly underestimated the pore size of the 2D hexagonal hole (And and Neimark, 2002; Liu D. et al., 2012). The diameter of the mesoporous material was evaluated by using the formula of the unit cell parameter. Which was closer to the size obtained from TEM (~ 3.76 nm).

**Table 1:** Element compositions, and textural parameters of OMC and NOMCs.

**Sample**	**C ^a^(%)**	**N ^a^(%)**	**H ^a^(%)**	**S_BET_^b^(m^2^·g^−1^)**	**V ^c^(cm^3^·g^−1^)**	**V ^d^(cm^3^·g^−1^)**	**D ^e^ (nm)**
OMC-750	88.56	0	3.84	651	0.33	0.19	3.04
NOMC-0.3-550	82.34	2.18	2.79	572	0.28	0.15	3.33
NOMC-0.3-650	84.71	2.05	2.67	619	0.30	0.17	3.15
NOMC-0.3-750	87.13	1.82	2.53	637	0.31	0.18	3.10
NOMC-0.3-850	89.17	1.71	2.37	653	0.30	0.18	3.04
NOMC-0.6-750	86.85	2.56	2.87	567	0.27	0.15	3.04
NOMC-0.9-750	84.97	3.08	2.46	449	0.26	0.13	2.79

a *Measured by combustion element analyses*.

b *The BET specific surface area*.

c *Total volume of the pore*.

d *Micropore volume*.

e *Pore size*.

**Figure 2:**
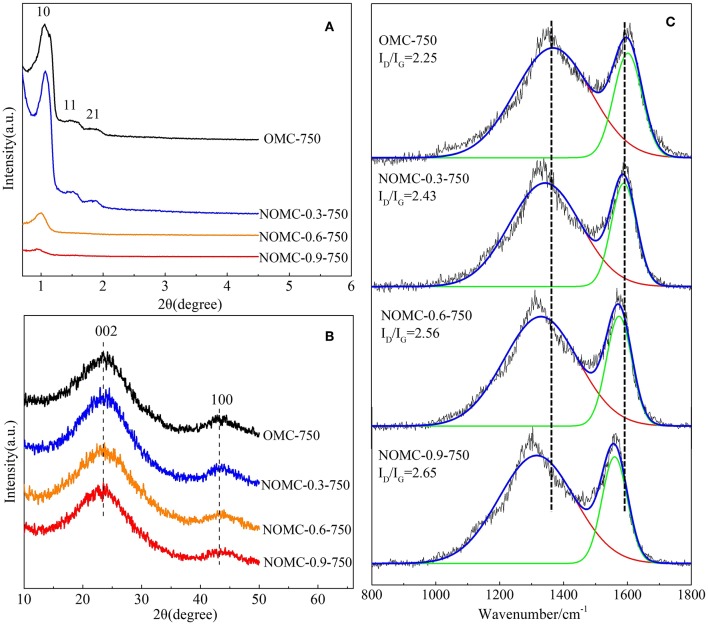
Small–angle **(A)**, wide–angle **(B)** XRD patterns and Raman spectra **(C)** of OMC and NOMCs, ordered mesoporous carbon with different nitrogen–doping amounts.

XRD measurements were performed to characterize the order of NOMCs and OMC. As depicted in Figure 2A, the small–angle XRD patterns of NOMCs with different nitrogen doping amounts showed at least one well–resolved diffraction peaks. The *d*–spacing ratios of these peaks was indexed as (10), (11), and (21) reflections associated with the 2D hexagonal p6m mesostructured. For highly nitrogen–doped samples, such as NOMC−0.6–750 and NOMC−0.9–750, the diffraction peaks were short and flat, indicating the disappearance of the mesostructured order. A wide–angle XRD spectrum could reveal information about the graphite properties of the sample (Figure 2B). The spectra of all of the samples had an obvious broadened reflection at 2θ = 23.8 and another relatively weak peak at 2θ = 43.8, which were assigned to diffractions from the (002) and (100) planes of the disordered graphitic carbon. The diffraction peak position and the peak shape of each sample were almost the same, indicating that the change of nitrogen doping content slightly affected the degree of graphitization of the sample.

The use of Raman spectroscopy has become an important tool for the analysis of carbon–based materials. In Figure 2C, like most sp^2^ carbon materials, all the samples in this study possess two characteristic Raman peaks at approximately 1320 cm^−1^ (D–band) and 1580 cm^−1^ (G–band). It is well accepted that the D–band is attributed to structural defects and disorders abduction with sp^3^ domains in carbon layers, whereas the G–band indicates the presence of crystalline graphite carbon (Chen et al., 2011; Ionescu et al., 2012). The intensity ratio of D–band to G–band, namely, I_D_/I_G_, is used to evaluate the degree of disorder within the carbon-based nanostructures (Dresselhaus et al., 2005). The higher I_D_/I_G_ ratio implies the higher defectiveness of graphite like layers by nitrogen doping. In addition, the Raman shift in the D–band and the G–band is redshifted with the increase of N content, which is probably because of the N doping leading to new chemical structures such as quaternary nitrogen and pyridine and the damage to OMC. The fitting of the spectrum reveals that NOMC−0.9–750 has the highest I_D_/I_G_, indicating that the incorporation of nitrogen reduces the crystallinity of the material, which is consisted with the results of XPS and XRD.

**Figure 3:**
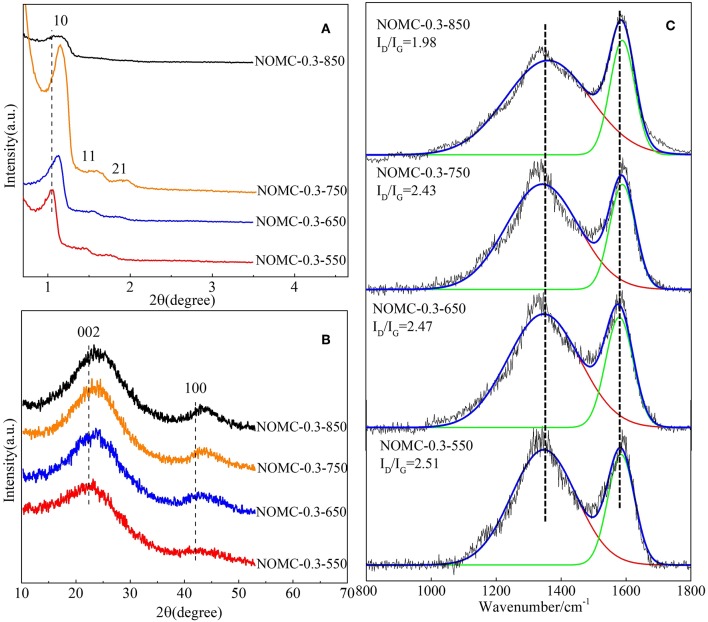
Small–angle **(A)** and wide–angle **(B)** XRD patterns and Raman spectra **(C)** of NOMCs, ordered mesoporous carbon with various pyrolysis temperatures.

To investigate the effect of pyrolysis temperature on the material structure, we performed XRD and Raman spectroscopy on the samples obtained at different pyrolysis temperatures. All of the samples except NOMC−0.3–850 have three clear diffraction peaks (Figure 3A), and the absence of NOMC−0.3–850 order may be related to the collapse of the channel at the highest temperature. As the pyrolysis temperature increases, the (10) peak slightly shifts to a higher angle, indicating the shrinkage of the mesoporous channels. The (002) diffraction peak (Figure 3B) of the samples obtained at a higher pyrolysis temperature is at a higher angle, suggesting that a higher pyrolysis temperature can enhance the graphitization of carbon. Figure 3C shows that all samples calcined at different temperatures possess the different I_D_/I_G_. The intensity ratios of D and G bands become small with the increase in carbonization temperature, but the value of I_D_/I_G_ becomes large when the carbonization temperature exceeds 850°C. The reason may be that the high carbonization temperature destroys the internal pore structure of NOMC samples, thereby decreasing the ordering degree of the NOMC samples.

**Figure 4:**
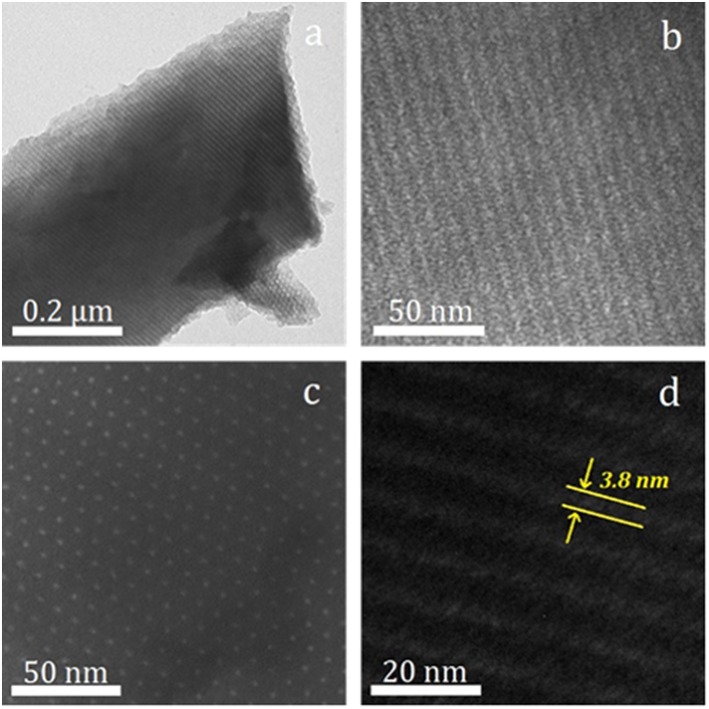
Various magnification TEM images of NOMC−0.3–750 viewed along [110] **(a,b,d)** and [001] **(c)** directions of hexagonal mesostructure.

Trace amounts of melamine incorporate rich N atoms into the carbon layer, but further increasing the amount of melamine does not continue this trend and hinders the formation of ordered channels. NOMC−0.3–750 is screened as the suitable support and further confirmed by observing the microscopic morphology through TEM. Figure 4 shows that the sample has an ordered hexagonal lattice sequence in the [001] direction and a parallel continuous stripe structure in the (110) direction, further confirming the 2D hexagonal mesoporous structure.

**Figure 5:**
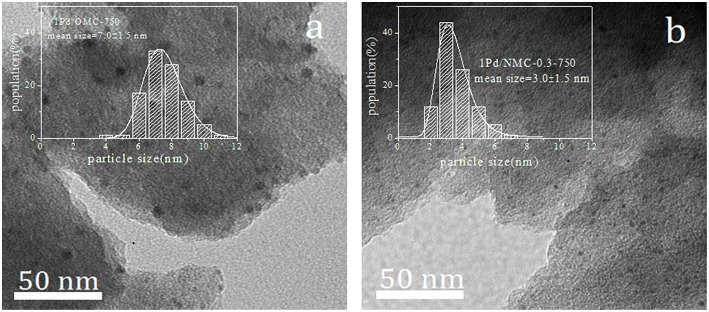
TEM micrographs and its corresponding Pd particles size distributions of NOMC–supported catalysts: 1Pd/OMC−750 **(a)**, 1Pd/NOMC−0.3–750 **(b)**.

We directly observed the mean particle size and its distribution for Pd/NOMCs catalysts by TEM (Figure 5). The particle size distribution is calculated by measuring ~100 particles on the corresponding TEM micrograph. Pd supported by OMC-750 (Figure 5a) is mainly present in the form of large-sized particles and distributed in pores and surfaces. However, after nitrogen is incorporated into the skeleton, Pd nanoparticles are uniformly dispersed and embedded in the mesoporous structure of the NOMCs, and the metal particles are effectively encapsulated by the channels of the NOMCs (Figure 5b). The fully enclosed Pd nanoparticles have a high catalytic activity for the selective oxidation of benzyl alcohol (Chen et al., 2010). Studies on the size dependence of nanoparticles have confirmed that metal support interaction is an important factor affecting the catalytic activity of the single metal catalyst (Comotti et al., 2004). The dispersion of metal particles on a carrier is closely related to the surface chemistry of a support. The free electrons provided by the N-containing species uniformly present in the carbon matrix to realize electron transfer with the Pd atoms during the adsorption, resulting in Pd mostly with the form of smaller particles.

**Figure 6:**
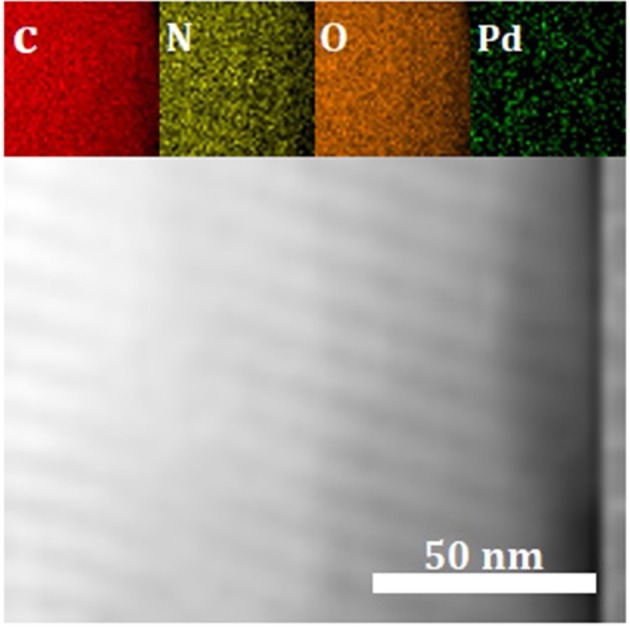
EDX maps of C, N, O and Pd elements for 1Pd/NOMC−0.3–750 according to the corresponding TEM image.

To obtain information regarding the elemental distribution of C, N, O, and Pd in the 1Pd/NOMC−0.3–750, we used a combination of a TEM and an EDX spectrometer to obtain an EDX maps (Figure 6). As the main elements of the material skeleton, C, N, and O atoms are evenly distributed, which shows that nitrogen is successfully doped into carbon materials. Figure 6 illustrates the uniform distribution of Pd nanoparticles on the surface and pores of NOMC-0.3-750, which is consistent with the TEM results.

**Figure 7:**
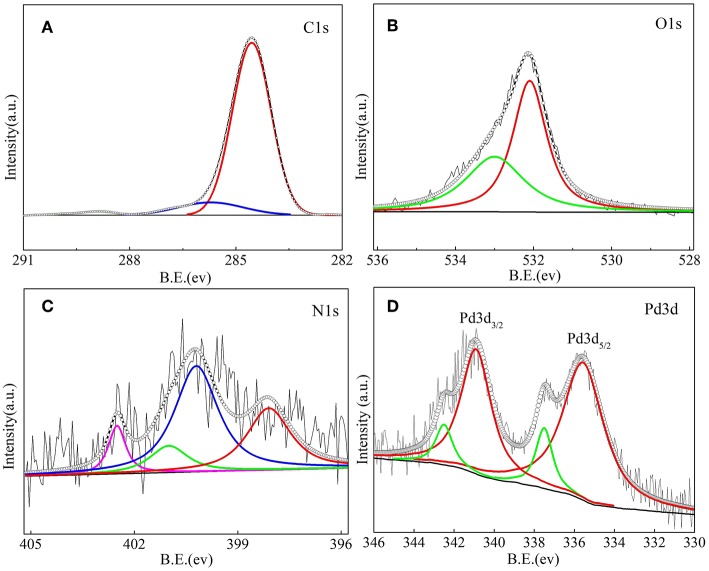
XPS of the C 1s core level **(A)**, O 1s core level **(B)** N 1s core level **(C)** and Pd 3d core level **(D)** of the 1Pd/NOMC−0.3–750 nanostructured material.

The surface chemical information of NOMCs can be obtained from the XPS spectrum shown in Figure 7. The peaks of C, N, and O are observed in the XPS spectra of NOMC−0.3–750, indicating that N is successfully doped into carbon materials. The N 1s spectrum (Figure 7C) can be divided into four peaks with bonding energies of 403.2 ± 0.1, 401.0 ± 0.1, 400.2 ± 0.1, and 398.1 ± 0.1 eV that respectively correspond to oxidized N, graphitic N, pyrrolic N, and pyridinic N because of different binding energies. The surface nitrogen content obtained from the XPS test for NOMC−0.3–750 is 1.56%, which is slightly lower than the nitrogen content 1.82% revealed through the elemental analysis. The C 1s spectra of NOMC−0.3–750 (Figure 7A) can be decomposed into three peaks. Two easily identifiable peaks at 284.5 and 285.7 eV correspond to C = C, and C–N or C–C functional groups, respectively. The weak peak near 288.9 eV can be attributed to C = N or C = O functional groups. The oxygen content of 7.16% mainly comes from the precursor. The O 1s XPS spectra can be deconvoluted into two peaks, namely, C = O (532.6 ± 0.1 eV) and C–OH (533.8 ± 0.1 eV). Figure 7D shows the fitting results of the XPS pattern of Pd 3d. The fitted peak of Pd 3d can be divided into two groups in which a set of characteristic peaks at 340.9 and 335.9 eV can be assigned to Pd metal particles. A relatively small pair of peaks at 342.5 eV for Pd 3d_3/2_ and 337.5 eV for Pd 3d_5/2_, indicates that some of the Pd elements exist as Pd^2+^. In the presence of basic sites such as pyridine nitrogen or pyrrole nitrogen, the reducibility of Pd can be decreased. Hence, a certain proportion of Pd^δ+^ exists. The XPS spectra of N 1s show that some of the N atoms exist in the form of a combination of pyridinic N, which can promote the anchoring of metal nanoparticles and the coordination of metal ions (Cui et al., 2016; Jiang et al., 2016).

### Catalytic Performance of Pd Catalysts

In the absence of solvent, Pd nanoparticles supported on the NOMCs are employed as a catalyst to selectively oxidize the benzyl alcohol to benzaldehyde and a small amount of benzyl benzoate. The main reactions involved in the oxidation of benzyl alcohol are as follows.

(1) $$\begin{array}{l} 2{\text{C}}_{6}{\text{H}}_{5}\text{C}{\text{H}}_{2}\text{OH}+{\text{O}}_{2}\to 2{\text{C}}_{6}{\text{H}}_{5}\text{CHO}+2{\text{H}}_{2}\text{O} \end{array}$$

(2) $$\begin{array}{l} 2{\text{C}}_{6}{\text{H}}_{5}\text{CHO}+{\text{O}}_{2}\to 2{\text{C}}_{6}{\text{H}}_{5}\text{COOH} \end{array}$$

(3) $$\begin{array}{l} {\text{C}}_{6}{\text{H}}_{5}\text{COOH}+{\text{C}}_{6}{\text{H}}_{5}\text{C}{\text{H}}_{2}\text{OH}\to {\text{C}}_{6}{\text{H}}_{5}\text{COOC}{\text{H}}_{2}{\text{C}}_{6}{\text{H}}_{5}+{\text{H}}_{2}\text{O} \end{array}$$

Currently, precious metals are still the common catalysts for the oxidation of benzyl alcohol. Hence, enhancing the catalytic efficiency and reducing the amount of precious metal used are the considerations for the synthesis of highly efficient catalysts. To explore suitable reaction conditions and to obtain the maximum yield of benzaldehyde, a number of comparative experiments were designed. The conversion of benzyl alcohol and the selectivity of benzaldehyde were obtained through GC analysis. However, it was found traces of benzoic acid in the GC analysis, indicating that at higher benzyl alcohol concentrations, the benzoic acid produced by reaction 2 was immediately esterified with benzyl alcohol to form benzyl benzoate (reaction 3).

To compare the effects of NOMC-0.3-750 and OMC-750 as a carrier on the catalytic performance, the oxidation reactions are performed under the same experimental conditions. In comparison with the conversion of 15.81% by using 1Pd/OMC-750, the catalytic performance of the catalyst 1Pd/NOMC-0.3-750 is obviously enhanced, the conversion is up to 24.63%, shown in Table 2. It is obvious that Pd nanoparticles supported by NOMC-0.3-750 has the highest TOF value (8696 h^−1^). Compared to OMC, NOMCs can provide rich anchoring sites to immobilize more monatomic Pd nanoparticles, which contributes to higher metal surface densities and conversion. The reduction of catalytic efficiency of 1Pd/NOMC-0.6-750 and 1Pd/NOMC-0.9-750 may be related to the reduction of order degree and surface area, because the ordered nitrogen-rich lattice structure has better mass transfer effect.

**Table 2:** Catalytic performance of NOMC Supported Pd catalysts with different N-doping amount^a^.

**Catalyst**	**Conversion (%)**	**Selectivity (%)**		**Benzaldehyde yield (%)**	**TOF (h^−1^)**
		**Benzaldehyde**	**Benzyl benzoate**		
1Pd/OMC−750	15.81	87.14	9.73	13.78	5022
1Pd/NOMC−0.3–750	24.63	85.71	13.56	21.11	8696
1Pd/NOMC−0.6–750	23.52	86.39	12.62	20.32	7901
1Pd/NOMC−0.9–750	20.13	86.05	12.44	17.32	6804

a *Reaction conditions: catalyst, 20 mg; benzyl alcohol, 50 mmol; O_2_, 20 mL/min; temperature, 160°C; reaction time, 3h*.

Under standard reaction conditions, 1Pd/NOMC−0.3–750 is used as a catalyst for the oxidation of benzyl alcohol at different temperatures (entries 1–4 in Table 3). It is noted that higher reaction temperatures lead to higher conversions, but the selectivity to benzaldehyde decreases. This phenomenon occurs probably because the molecular oxygen reacts further with benzaldehyde to produce phenylacetate, which can be rapidly esterified with benzyl alcohol to form benzyl benzoate at high temperatures, thereby decreasing the selectivity of benzaldehyde. Nevertheless, benzaldehyde is the main product of the catalytic oxidation of benzyl alcohol under all reaction conditions.

**Table 3:** Catalytic properties of Pd-containing catalysts supported on NOMC-0.3–750^a^.

**Entry**	**Catalyst**	**Temperature (^°^C)**	**Conversion (%)**	**Selectivity (%)**		**TOF (h^−1^)**
				**Benzaldehyde**	**Benzyl benzoate**	
1	1Pd/NOMC−0.3–750	70	1.19	96.12	2.93	420
2	1Pd/NOMC−0.3–750	100	5.27	94.76	4.89	1,861
3	1Pd/NOMC−0.3–750	130	13.41	92.51	6.68	4,734
4	1Pd/NOMC−0.3–750	160	24.63	85.71	13.56	8,698
5	0.5Pd/NOMC−0.3–750	160	22.78	86.10	12.62	8,041
6	2Pd/NOMC−0.3–750	160	16.5	84.79	13.95	6,495

a *Reaction conditions: benzyl alcohol/Pd = 250 mol/g; O_2_, 20 mL/min^−1^; temperature, 160 °C; reaction time, 3h*.

Pd loading is an important factor affecting the catalytic efficiency of the catalyst. Entries 4–6 in Table 3 show the catalytic results of NOMC−0.3–750 with different Pd loadings (0.5–2 wt.%). As Pd loading increases from 0.5 to 1 wt.%, the benzyl alcohol conversion increases as the number of available active sites participating in the reaction increases. After Pd load is further increased to 2 wt.%, the conversion decreases slightly possibly because of the decrease in the number of available reactive centers. As the Pd content is high, the agglomeration of nanoparticles will occur (Li et al., 2008). In addition, the effect of Pd/NOMC mass ratio on selectivity is weak under current reaction conditions. Other studies have revealed similar findings (Haider et al., 2008).

**Figure 8:**
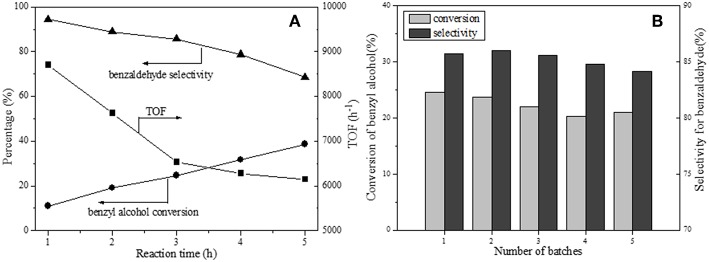
Time course **(A)** and recycling ability **(B)** of 1Pd/NOMC-0.3-750 for benzyl alcohol oxidation. Reaction conditions: catalyst, 20 mg; benzyl alcohol, 50 mmol; O_2_, 20 mL/min^−1^; temperature, 160°C; reaction time, 3 h.

The 1 wt.% Pd load has been verified to be the most suitable amount. The time course of 1Pd/NOMC−0.3–750 for benzyl alcohol oxidation is monitored regularly as depicted in Figure 8A. It is noted that the conversion of benzyl alcohol increases monotonously with time, but the growth rate slows down, which is more intuitively reflected in the reduction of TOF value. The decrease in the selectivity of benzaldehyde is attributed to its further oxidation to benzoic acid, which can be rapidly esterified with benzyl alcohol to form benzyl benzoate without the intervention of a catalyst (Enache et al., 2007).

Catalytic stability is an important indicator of a catalyst. The leaching of low–stability metal particles during the reaction is an important factor in reducing the activity of a catalyst. To eliminate the effect of the loss of the catalyst mass on the results, we maintained the total amount of the catalyst at 20 mg by adding the catalyst used for each recovery. Figure 8B shows the recycling capacity of 1Pd/NOMC−0.3–750 for five cycles. The cycle test demonstrates that the conversion slightly decreases, and the selectivity remains stable. This observation verifies that no significant leaching of Pd nanoparticles occurs, and the developed catalyst has a good stability.

## Conclusions

NOMCs were synthesized via a one–pot hydrothermal route, which were provided with a 2D hexagonal mesoporous structure. In comparison with traditional template methods, including soft and hard templates, the present method had a simple preparation process and a short synthesis period. The structural characterization revealed that NOMCs could anchor the Pd nanoparticles to enhance its uniform dispersion, which was favorable to the activity and stability in the catalytic reaction. The developed 1Pd/NOMC-0.3-750 catalyst showed excellent catalytic performance for solvent free oxidation of benzyl alcohol, its TOF value was up to 8698 h^−1^ and maintained a good catalytic stability after 5 cycles of the reaction. The proposed catalyst has a potential engineering application value.

## Data Availability

The raw data supporting the conclusions of this manuscript will be made available by the authors, without undue reservation, to any qualified researcher.

## Author Contributions

HS and HG designed the experiments. ZL and YW carried out the experiments. LQ and CZ analyzed the experimental results. XY assisted and analyzed the sequencing data. HS and MX wrote the manuscript.

### Conflict of Interest Statement

The authors declare that the research was conducted in the absence of any commercial or financial relationships that could be construed as a potential conflict of interest.
